# Mechanisms of allergen-specific immunotherapy

**DOI:** 10.1186/2045-7022-2-2

**Published:** 2012-01-05

**Authors:** Hiroyuki Fujita, Michael B Soyka, Mübeccel Akdis, Cezmi A Akdis

**Affiliations:** 1 Swiss Institute of Allergy and Asthma Research (SIAF), University of Zurich, Davos, Switzerland; 2 Christine Kühne-Center for Allergy Research and Education (CK-CARE), Davos, Switzerland; 3 Department of Environmental Immuno-Dermatology, Yokohama City University Graduate School of Medicine, Yokohama, Japan

## Abstract

Allergen-specific immunotherapy (allergen-SIT) is a potentially curative treatment approach in allergic diseases. It has been used for almost 100 years as a desensitizing therapy. The induction of peripheral T cell tolerance and promotion of the formation of regulatory T-cells are key mechanisms in allergen-SIT. Both FOXP3^+^CD4^+^CD25^+ ^regulatory T (Treg) cells and inducible IL-10- and TGF-β-producing type 1 Treg (Tr1) cells may prevent the development of allergic diseases and play a role in successful allergen-SIT and healthy immune response via several mechanisms. The mechanisms of suppression of different pro-inflammatory cells, such as eosinophils, mast cells and basophils and the development of allergen tolerance also directly or indirectly involves Treg cells. Furthermore, the formation of non-inflammatory antibodies particularly IgG4 is induced by IL-10. Knowledge of these molecular basis is crucial in the understanding the regulation of immune responses and their possible therapeutic targets in allergic diseases.

## Background

The immune system is a complex interactive network with the capacity of protecting the host from a number of pathogens while keeping a state of tolerance to self and innocuous non-self antigens. Allergy is one of the immune tolerance-related diseases that arises as a direct consequence of a dysregulated immune response. Currently, allergen-specific immunotherapy (allergen-SIT) by the administration of increasing doses of allergen extracts remains the single curative approach to allergic diseases with the potential to modify its course [1,2]. The aim of this review is to discuss the mechanism of allergen-SIT and the current clinical and experimental evidence in the field of immune tolerance induction in allergic diseases.

### Pathogenesis of allergic diseases

Allergic diseases represent complex innate and adaptive immune responses to environmental antigens leading to inflammatory reactions with a T-helper-2-type cell and allergen-specific IgE predominance [3,4]. CD4^+ ^Naïve T cells differentiate into distinct T cell subsets such as Th1, Th2, Th9, Th17 and Th22 type memory and effector cells depending on the cytokines, other molecules and cells present in the microenvironment [5]. Once a Th2 shift is established, the mechanism of allergic diseases consists of two main phases. In the early phase sensitization and the development of memory cells takes place. The late phase is characterized by inflammation and tissue injury caused by effector cell action. During the sensitization phase, the differentiation and clonal expansion of allergen-specific CD4^+ ^Th2 cells, with the capability of producing IL-4 and IL-13, are essential in the induction of class switching to the ε immunoglobulin heavy chain in B cells and the production of allergen-specific IgE antibodies. Allergen-specific IgE binds to the high affinity receptor FcεRI, on the surface of mast cells and basophils as well as to antigen presenting cells (APCs), which in turn allows for an increased uptake of allergens [6]. The engagement of IgE on effector cells leads to the sensitization of the patients to a specific allergen [7]. Upon re-exposure receptor-bound IgE molecules are crosslinked, which in turn results in the activation and release of mediators that cause[8] the development of type I hypersensitivity reactions [9,10].

During the development of allergic diseases, effector Th2 cells not only produce traditional Th2 cytokines such as IL-4, IL-5, IL-9 and IL-13 [11,12], but also novel cytokines with proinflammatory functions, such as IL-25, IL-31 and IL-33 [13-19]. These cytokines induce allergen-specific IgE, eosinophilia, mucus production and the recruitment of inflammatory cells to inflamed tissues. Predominance of Th2 cells might be caused by an increased tendency to activation-induced cell death of high IFN-γ-producing Th1 cells as it is commonly observed in patients with atopic disorders [20]. Th1 cells also play a role in the effector phase of allergic diseases by inducing apoptosis of epithelial cells and/or smooth muscle cells in asthma and keratinocytes in atopic dermatitis [21-25]. In vitro, the suppressive capacity of CD4^+^CD25^+ ^T-regulatory (Treg) cells from hay fever patients is decreased during the pollen season [26]. Allergen-specific IL-10 secreting Treg cells were shown to be decreased in blood obtained from patients with persistent allergic rhinitis although the number and function of CD4^+^CD25^+ ^Treg cells were normal [27]. Different symptomatic treatments like antihistamines, leukotriene receptor antagonists and glucocorticoids are used in allergic diseases, however do not provide the possibility of cure [6]. Glucocorticoids, systemically applied, increases the frequency of CD25^+ ^memory CD4^+ ^T cells and FOXP3 messenger RNA [28].

### Mechanisms of allergen-specific immunotherapy

#### T cell regulation

**Figure 1:**
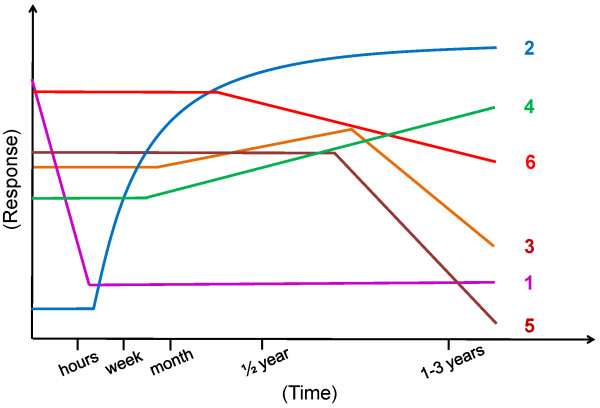
**Immune regulation during the time course of allergen-SIT**. Specific immune responses are observed during the course of allergen-SIT. 1. An early desensitization effect including decrease in mast cell and basophil degranulation soon after the first administration of allergens. 2. Generation of allergen-specific Treg cells and suppression of effector cells. 3. An early increase and a late decrease in specific IgE levels. 4. A relatively early increase in specific IgG4. 5. A late decrease in type I skin test reactivity. 6. A decrease in tissue mast cell and eosinophil numbers and a release of their mediators after a few months.

**Figure 2:**
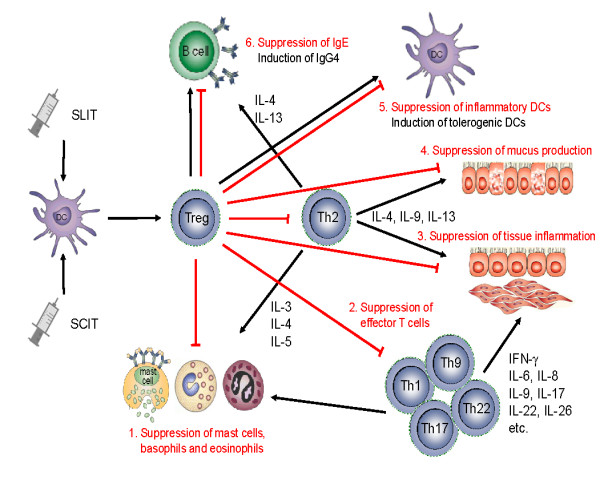
**Mechanisms of allergen-specific immunotherapy and the role of regulatory T cells in allergic diseases**. An allergen is taken up by regional dendritic cells leading to the induction of regulatory T cells. These cells suppress allergic responses directly and indirectly by the following mechanisms. 1. Suppression of mast cells, basophils and eosinophils. 2. Suppression of effector T cells. 3. Suppression of inflammatory cell migration to tissues and tissue inflammation. 4. Suppression of mucus production. 5. Suppression of inflammatory dendritic cells and induction of tolerogenic dendritic cells. 6. Suppression of allergen-specific IgE and induction of IgG4 from B cells.

Since allergic diseases are not only Th2 driven, but much rather form complex immune disorders, the aim of allergen SIT is to induce the peripheral T cell tolerance, modulate the thresholds for mast cell and basophil activation and decrease IgE-mediated histamine release [29] (Figure 1 and 2). The induction of peripheral T cell tolerance represents an essential step in allergen-SIT. Peripheral T cell tolerance is characterized by the generation of allergen-specific Treg cells that are able to produce anti-inflammatory cytokines such as IL-10 and TGF-β. Multiple mechanisms are involved in the suppression and/or control of allergic inflammation. Treg cells not only diminish Th2 immune responses, but also target other cell types such as DCs, mast cells, basophils and eosinophils. Treg cells regulate allergen-specific-IgE and are capable of inducing IgG4 and IgA production [30-33]. Treg cells are able to directly inhibit mast cell degranulation by OX40-OX40Ligand interaction [30]. There are two main Treg cells subsets with distinct phenotypes and mechanisms of action. One is the naturally occurring, thymic selected FOXP3^+^CD4^+^CD25^+ ^Treg cells. The other subset is referred to as the inducible Treg cells, generated in the periphery under tolerogenic conditions. The two subsets of inducible Treg cells, namely the FOXP3^+ ^and the IL-10-positive Tr1 cells play a key role in allergen tolerance and they can be induced by allergen SIT in humans [34,35].

It is well established that FOXP3 acts as the main transcription factor for Treg cells development and function [36]. FOXP3 mutations in humans lead to the X-linked immune dysregulation polyendocrinopathy enteropathy syndrome [37]. Patients affected by this defect suffer from allergic and autoimmune diseases due to non-functional Treg cells. FOXP3 mutations in mice lead to the spontaneous development of lymphoproliferative disease, allergic airway inflammation, hyper IgE syndrome, eosinophilia and autoimmune diseases [38]. It has been demonstrated that FOXP3 directly interacts with the Runt-related transcription factor 1 (RUNX1), which reduces IL-2 and IFN-γ expressions and exerts suppressive functions [39]. A recent study in mice has also shown that the RUNX transcription factors are essential for maintaining high FOXP3 expression and confirm Treg lineage identity [40]. Moreover, a novel molecular mechanism defining Runx transcription factors as a linking molecule in TGF-beta induced Foxp3 expression in Treg differentiation and function was shown [41].

Several studies have demonstrated that allergen-specific Tr1 cells are highly present in healthy individuals to prevent unwanted immune response to nonpathogenic environmental antigens [42-45]. The three different allergen-specific T cell subsets Th1, Th2 and Tr1 that recognize the same T-cell epitopes co-exist in both healthy and allergic individuals in different proportions. Persons with high numbers of Th2-cells are prone to develop an allergic phenotype, whereas Tr1 predominance seems rather protective in this perspective [43]. High dose-allergen exposure and the induction of tolerance have been well investigated for bee venom and cat allergens [45]. Beekeepers face high levels of bee venom antigens during the beekeeping season. Repeated exposure to the venom allergens results in a reduction in T-cell-related cutaneous late-phase reactions and an impaired capacity of allergen-specific T cells to proliferate and produce Th1 and Th2 cytokines. This reaction persists as long as bee venom exposure continues. Venom-specific T cell proliferation, which is suppressed at the time of exposure returns to initial levels within several months after the end of the beekeeping season. This phenomenon correlates with a clonal switch of venom antigen-specific Th1 and Th2 cells toward IL-10-secreting Tr1 cells. In this model, histamine receptor 2 is up-regulated on specific Th2 cells and plays a dual role in the suppression of allergen-stimulated T cells and in the induction of IL-10 production. Allergen-specific IgG4/IgE ratios are about thousand times higher in non-allergic beekeepers compared with bee venom allergic individuals [46]. In another high-dose allergen exposure model with cat allergens, an increase of allergen-specific IgG4 and IL-10-producing Tr1 cells were demonstrated [47]. IL-10 reveals multiple ways of action in allergen tolerance. It leads to the downregulation of MHC-II molecules on APCs and inhibits a wide range of proinflammatory cytokines and cytokine receptors [48]. IL-10 reduces IL-5 production by Th0 and Th2 and downregulates eosinophil activity [49]. Thus, reduced levels of eosinophilic cationic protein were also found during SIT [50]. Treg cells derived TGF-β bears a great potential in allergen tolerance. This cytokine not only inhibits B-cell proliferation and differentiation, but also decreases immunoglobulins with the exception of mucosal IgA [51,52]. TGF-β is able to promote further CD4^+^CD25^+ ^T cell conversion from naïve CD4^+^CD25^- ^T cells [53]. SIT is able to increase TGF-β production and is therefore associated with higher amounts of specific IgA [44]. Furthermore Treg cells are capable of downregulating costimulatory molecules on DCs and compete with naïve T cells by creating aggregates around DCs, thus inhibiting their maturation [54].

Apart from the two mentioned main subsets of Treg cells, several other T cells with regulatory function have been demonstrated. CD8^+^CD28^- ^T cells showed suppressor capacity **in vitro**. They are able to prevent upregulation of B7 molecules induced by helper T cells on professional APCs and play role in oral tolerance [55,56]. TCRαβ^+^CD4^-^CD8^- ^double-negative Treg cells have been shown to suppress antigen-specific immune responses mediated by CD4^+ ^T and CD8^+ ^T cells in humans and mice [57]. NKreg cells have the capacity to abort antigen-specific T cell responses [58]. A certain subset of invariant NKT cells also possesses control functions. The combination of IL-27 and IFN-γ produced by invariant natural killer T (iNKT) cells suppresses the established Th2 functions in mice [59]. Antigen-containing liposomal α-galactosylceramide, which is a representative ligand for iNKT cells, lowers antigen-specific IgE via the induction of tolerogenic DCs and Treg cells [60].

#### Regulation of allergen-specific antibodies

IgG4 is a non-inflammatory isotype protecting from allergic reaction. It is thought to capture the allergen before reaching the effector cell-bound IgE and thus to prevent the activation of mast cells and basophils [29]. IgG4 is unable to bind complement efficiently and contains two different antigen-binding sites on one molecule. The bi-specificity turns the antibody functionally monovalent, thus preventing it from forming complexes [61]. Allergen-specific IgG4 might be directed against different epitopes of the allergen than IgE, yet an inhibition of the IgE-allergen binding by certain IgG is observed resulting in a blocking effect [62]. Successful SIT is associated with an increase in IgG-blocking activity that is not solely dependent on the quantity of IgG antibodies [63,64]. It seems to be relevant rather to measure the blocking activity & affinity of specific IgG and its subsets (i.e. IgG4, IgG1), instead of their levels in sera. The induction of IgG also plays a role both by the inhibition of IgE-facilitated antigen presentation and the inhibition of IgE-mediated release of mediators from mast cells and basophils [64]. Allergen-SIT induces a transient increase of specific IgE levels in serum, followed by a gradual decrease over months or years of treatment [65]. Serum IgE levels cannot explain the diminished responsiveness to a specific allergen, because the decrease of serum IgE levels is relatively late and does not correlate with clinical improvement after SIT. The decrease in IgE/IgG4 ratio during allergen SIT seems to be a feature of skewing from allergen-specific Th2 to Treg cell predominance. Since the class switching of IgG4 is caused by the co-stimulation with IL-4 and IL-10, IL-10 decreases IL-4-induced IgE switching but increases IL-4-induced IgG4 production. Thus, IL-10 not only generates tolerance in T cells, but also regulates the allergen-specific antibody isotype formation toward a non-inflammatory direction.

### Novel suppressive cell subsets and cytokines

Recently, several novel suppressive cell subsets and cytokines have been demonstrated. They could form targets for novel allergen-specific therapies and need to be included in the future research on SIT.

#### Regulatory B cells

B cells are the only cell type that is capable of producing antibodies and therefore are the central cellular component of the humoral immune responses. In addition, B cells can modulate CD4^+ ^T cell responses by presenting antigens, expressing costimulatory molecules or producing cytokines [66]. Regulatory B (Breg) cells, which are able to secrete IL-10, regulate the development, proliferation and maintenance of CD4^+ ^T effector and memory T cells as well as Treg cells [67]. Recent studies suggest that there are several phenotypically distinct populations of IL-10-producing Breg cells [68-72]. Transitional 2-marginal zone precursor B cells which express CD1d^hi^CD21^hi^CD23^+^IgM^+^, follicular B cells and B cells expressing high levels of CD1d can produce IL-10 and play a role as Breg cells [73-76]. Among several Breg cell subsets, CD1d^hi^CD5^+^CD19^hi ^Breg cells are well studied [77]. The transfer of this subset prevents CD4^+ ^T cell-dependent contact hypersensitivity in mice. Since this suppressive function is antigen dependent, Breg cells from mice primed with an antigen were not able to suppress the T cell inflammation elicited by another antigen. Moreover, this function requires their ability to produce IL-10. The Breg cell subset is also characterized in human blood as CD24^hi^CD27^+ ^B cells [78]. They can negatively regulate monocyte cytokine production via IL-10-dependent pathways. Taken together, the antigen-specific Breg cell subset can be a potent candidate for novel allergen-specific immunotherapies.

### IL-35

IL-35 is a heterodimeric cytokine consisting of EBI3 and the p35 subunit of IL-12 [79]. In mice, IL-35 is constitutively secreted by FOXP3^+ ^Treg cells [80]. The expression of EBI3 and p35 in FOXP3^+ ^Treg cells is higher than in effector T cells and transcription analysis identifies EBI3 as a downstream target of FOXP3 [81]. Although IL-35 does not affect the FOXP3 expression on Treg cells, it can induce the IL-10 production in CD4^+^CD25^+ ^Treg cells. Stimulation of CD4^+^CD25^- ^effector T cells by IL-35 and anti-CD3/anti-CD28 antibodies induces proliferation of these, enhances IFN-γ production and up-regulates the transcription factor T-bet on T cells [82]. CD4^+^CD25^+ ^T cells expanded in the presence of IL-35 are able to suppress the proliferation of CD4^+^CD25^- ^T cells. IL-35, but not EBI3 alone, inhibited the differentiation of CD4^+ ^T cells into Th17 cells. IL-35 also increases serum levels of IL-10 and IFN-γ whereas it decreases IL-17 [82]. Furthermore, treatment of naïve T cells with IL-35 induces a novel regulatory T cell subset, which mediates suppression via IL-35 but not via other Treg related cytokines such as IL-10 or TGF-β [83].

### Clinical use

Allergen-SIT has been used for more than 100 years in the therapy of allergic diseases. Desensitization represents a potentially curative and specific approach to allergies [29]. Although sublingual immunotherapy (SLIT) and subcutaneous immunotherapy (SCIT) are the two main routes of administration, SLIT seems to be the more safe and favorable route of both. Several large-scaled, randomized, double-blinded, placebo-controlled trials demonstrated the long lasting and disease-modifying effects of SLIT [84-88]. Oral SIT possesses a high potential for the development of novel treatment modalities. In fact such approaches including oral immunotherapy for food allergy are under development [89-93].

SLIT depends on anatomical and functional characteristics of the oral mucosal tissue, which has a natural tolerogenic character. It possesses rapid wound healing capabilities with little scar formation and defies inflammation in spite of a high bacterial colonization. The lack of inflammatory cells around mucosal tissue and a high permeability for allergens enable efficient sublingual immunotherapy [94]. The initial step in SLIT is the uptake of an allergen by Langerhans cells within the mucosa via the high affinity surface IgE receptors [95,96]. This leads to the production of IL-10 and induction of T cells with a regulatory phenotype **in vitro **[97]. The mechanisms of action in SLIT have been found to be similar as in injection immunotherapy: Sublingual FOXP3-expressing cells are promoted, allergen-specific IgG4 and IgA is increased and the inhibitory activity on IgE was found to be enhanced in a time dependent manner [98].

Although clinical trials of allergen-SIT demonstrate treatment efficacy in various allergic diseases such as allergic asthma, allergic rhinitis, stinging insect hypersensitivity and aero-allergen-induced atopic dermatitis, there is a risk of serious adverse reactions, which can be classified in two categories: local reactions appearing as erythema, pruritus and swelling at the injection site of SCIT; and systemic reactions appearing as anaphylaxis from mild to serious life-threatening severity [99-101].

Such side effects are one of the difficulties of SIT to be overcome. Allergen extract-based SIT includes the risk of anaphylactic side effects and the potential to induce novel sensitization to proteins from vaccines [102]. On the other hand, it might be difficult to standardize such vaccines leading to inconsistent results in SIT. Current research focuses on these problems by creating recombinant vaccines [102] and by altering the route of administration. Modification of recombinant vaccines by fusion with so called modular antigen transducer proteins may enhance specific antibody production and is a potential way of reducing the amount of vaccine needed and therefore limiting side effects [103]. The administration of a vaccine by intralymphatic injection is also a promising way of reducing the amount of the allergen dose and therefore improving safety [104].

Systemic side effects are known to occur in the initial phase of desensitization. Patients need to be monitored during this period. Different time regimes are used for the administration of the first vaccinations. Currently ultra-rush procedures are efficiently used in hymenoptera allergy SIT [105]. An increase in Treg cells along with a Th2 to Th1 switch has been shown to occur already during the first 24 h [106]. This type of time regime is now also being investigated in SLIT [107].

## Conclusions

Recent developments on molecular mechanisms of immune regulation in the area of allergy have provided substantial knowledge on allergen-tolerance. The induction of peripheral T cell tolerance by Treg cells is a key point in the suppression of allergic inflammation. FOXP3^+^CD4^+^CD25^+ ^Treg cells and Tr1 cells secrete suppressive cytokines such as IL-10 and TGF-β, and lead to the production of non-inflammatory antibody subtypes such as IgG4 and IgA. Novel suppressive cell subsets and cytokines such as Breg cells and IL-35 may form targets for new SIT approaches. The understanding of the molecular processes enables us to better understand the regulation of the immune response. Novel vaccines are expected to shorten the duration, decrease the side effects and increase the efficiency of the treatment. Targeting the newly identified molecules could not only improve current anti-allergic therapies, but might also help to treat other immune-related disorders such as autoimmunity, organ transplant rejection, malignant neoplasms and various types of infections.

## List of abbreviations used

Breg: regulatory B; Cs: Dendritic cells; FOXP3: Forkhead box protein 3; iNKT: invariant natural killer T; RUNX: Runt-related transcription factor; SCIT: Subcutaneous immunotherapy; SIT: Specific immunotherapy; SLIT: Sublingual immunotherapy; Tr1: T regulatory type 1; Treg: regulatory T

## Competing interests

The authors declare that they have no competing interests.

## Authors' contributions

HF wrote the manuscript. MS revised and wrote the manuscript. MA revised the manuscript. CA wrote and revised the manuscript. All authors read and approved the final manuscript.

## Authors' information

HF is a research fellow at the Swiss Institute of Allergy and Asthma Research (SIAF) and an assistant professor of department of Environmental Immuno-Dermatology, Yokohama City University Graduate School of Medicine. MS is a research fellow at the SIAF. MA is the head of immunodermatology group of SIAF. CA is a director of SIAF, one of the directors of Christine Kühne - Center for Allergy Research and Education (CK-CARE) and president of the European Academy of Allergy Clinical Immunology (EAACI).
